# The ONDRISeq panel: custom-designed next-generation sequencing of genes related to neurodegeneration

**DOI:** 10.1038/npjgenmed.2016.32

**Published:** 2016-09-21

**Authors:** Sali M K Farhan, Allison A Dilliott, Mahdi Ghani, Christine Sato, Eric Liang, Ming Zhang, Adam D McIntyre, Henian Cao, Lemuel Racacho, John F Robinson, Michael J Strong, Mario Masellis, Peter St George-Hyslop, Dennis E Bulman, Ekaterina Rogaeva, Robert A Hegele, ONDRI Investigators

**Affiliations:** 1 Robarts Research Institute, Schulich School of Medicine and Dentistry, Western University, London, ON, Canada; 2 Department of Biochemistry, Schulich School of Medicine and Dentistry, Western University, London, ON, Canada; 3 Tanz Centre for Research in Neurodegenerative Diseases, University of Toronto, Toronto, ON, Canada; 4 Department of Microbiology and Immunology, Faculty of Medicine, Department of Biochemistry, University of Ottawa, Ottawa, ON, Canada; 5 Children's Hospital of Eastern Ontario Research Institute, Ottawa, ON, Canada; 6 Department of Clinical Neurological Sciences, Schulich School of Medicine & Dentistry, Western University, London, ON, Canada; 7 Department of Medicine (Neurology), Sunnybrook Health Sciences Centre, LC Campbell Cognitive Neurology Research Unit, Hurvitz Brain Science Research Program, Sunnybrook Research Institute, University of Toronto, Toronto, ON, Canada; 8 Department of Clinical Neurosciences, Cambridge Institute for Medical Research, University of Cambridge, Cambridge, UK; 9 Department of Pediatrics, Faculty of Medicine, University of Ottawa, Ottawa, ON, Canada

## Abstract

The Ontario Neurodegenerative Disease Research Initiative (ONDRI) is a multimodal, multi-year, prospective observational cohort study to characterise five diseases: (1) Alzheimer’s disease (AD) or amnestic single or multidomain mild cognitive impairment (aMCI) (AD/MCI); (2) amyotrophic lateral sclerosis (ALS); (3) frontotemporal dementia (FTD); (4) Parkinson’s disease (PD); and (5) vascular cognitive impairment (VCI). The ONDRI Genomics subgroup is investigating the genetic basis of neurodegeneration. We have developed a custom next-generation-sequencing-based panel, ONDRISeq that targets 80 genes known to be associated with neurodegeneration. We processed DNA collected from 216 individuals diagnosed with one of the five diseases, on ONDRISeq. All runs were executed on a MiSeq instrument and subjected to rigorous quality control assessments. We also independently validated a subset of the variant calls using NeuroX (a genome-wide array for neurodegenerative disorders), TaqMan allelic discrimination assay, or Sanger sequencing. ONDRISeq consistently generated high-quality genotyping calls and on average, 92% of targeted bases are covered by at least 30 reads. We also observed 100% concordance for the variants identified via ONDRISeq and validated by other genomic technologies. We were successful in detecting known as well as novel rare variants in 72.2% of cases although not all variants are disease-causing. Using ONDRISeq, we also found that the *APOE* E4 allele had a frequency of 0.167 in these samples. Our optimised workflow highlights next-generation sequencing as a robust tool in elucidating the genetic basis of neurodegenerative diseases by screening multiple candidate genes simultaneously.

## Introduction

Dementia encompasses a heterogeneous group of neurodegenerative diseases characterised by a progressive decline in cognitive function, language deficiency, and in some cases, motor impairment and behavioural anomalies. Currently, dementia has a global prevalence of 47.5 million cases and an incidence of 7.7 million new cases annually.^1–3^ Although today there are no direct treatments available to alter the progressive disease course, early diagnosis has been one of the best predictors of disease outcome.^3,4^ Further understanding of the molecular basis of dementia can lead to earlier diagnosis and the eventual development of targeted and efficacious treatment modalities.

Our group is part of the Ontario Neurodegenerative Disease Research Initiative (ONDRI), a multimodal, multi-year, prospective observational cohort study designed to address the effect of small vessel disease in neurodegeneration. ONDRI is recruiting ~600 participants diagnosed with one of the following five diseases: (1) Alzheimer’s disease (AD) or amnestic single- or multidomain mild cognitive impairment (aMCI) (AD/MCI); (2) amyotrophic lateral sclerosis (ALS); (3) frontotemporal dementia (FTD); (4) Parkinson’s disease (PD); and (5) vascular cognitive impairment (VCI).

Genetics is an important risk factor for neurodegenerative disease. Approximately 5–10% of cases with neurodegenerative diseases are familial and can be attributed to several genes.^5–7^ However, it is likely we are underestimating the incidence of familial cases based on clinical ascertainment, as the death of presymptomatic individuals may be due to other medical or extrahealth incidents prior to the development of the neurodegenerative syndrome. Furthermore, genetic testing is not universally recommended in the clinical management guidelines of neurodegenerative diseases.^8–13^ As such, most neurologists, if they choose to pursue genetic testing, only screen for a small subset of genes and often choose to genotype their patients for highly penetrant and known variants rather than agnostically sequencing all neurodegenerative disease genes. Together, these common clinical ascertainments as well as the high costs associated with genetic testing skew the incidence rates to significantly less than what is perhaps biologically accurate. The five neurodegenerative disorders under study could partly be caused by single, rare, pathogenic variants (monogenic) or multiple, small effect variants acting synergistically to mediate disease expression (oligogenic).

Advancements in next-generation sequencing (NGS) have allowed for efficient genetic variant detection at reduced costs. Currently, there are three main types of NGS applications including: (1) whole-genome sequencing (WGS); (2) whole-exome sequencing (WES); and (3) targeted gene panels.^14^ WGS is an indiscriminate approach that evaluates the genetic information in an individual’s entire genome. In contrast, WES targets only the protein-coding regions of the genome as disease-associated variants are significantly over-represented in coding regions.^14^ Consequently, WES has been one of the most widely used NGS approaches, however it still presents with several challenges. First, the cost of WES with adequate coverage (i.e., minimum ×30) still remains high at approximately $700. This makes the cumulative cost for studies with a large sample size prohibitively expensive. Second, the amount of genetic variation generated from the exome is excessive and often overwhelming for many researchers and more so for clinicians who may require the patient’s genetic diagnosis to determine whether any genotype-specific treatments are available. Third, WES can generate secondary findings unrelated to the disease of interest, which should be reported to the patient’s primary healthcare provider, in accordance with the guidelines proposed by the American College of Medical Genetics.^15^ Thus, in both clinical and research applications, WGS or WES data are still often reduced to focus on likely pathogenic disease-specific loci. In contrast, the use of a targeted gene panel that is clinically focused on the genes underlying the disease(s) of interest, overcomes these issues that often arise when sifting through WGS and WES data.

Herein, we describe the development of a NGS based custom-designed resequencing neurodegeneration gene panel, which we have used to identify genetic variants in neurodegenerative disease cases. ‘ONDRISeq’ allows the screening of patients for variants in 80 genes implicated in neurodegenerative and cerebrovascular disease pathways. However, analysis of 80 genes can still yield an excess of genetic variation. We dichotomised all clinically relevant variants from those of uncertain significance using our integrated custom bioinformatics workflow. Our application of NGS in complex, multifactorial disorders has the potential to identify disease-specific risk markers and potentially, overlapping pathways common across all five diseases.

## Results

### Study subjects

We recruited 216 participants affected with one of the following disorders: (1) AD/MCI, *n*=40; (2) ALS, *n*=22; (3) FTD, *n*=21; (4) PD, *n*=56; and (5) VCI, *n*=77 as part of the ONDRI study (Table 1). The average age of our participants was 69.4±7.8 years. Not surprisingly, individuals diagnosed with ALS were the youngest in our cohort with an average age of 61.9±9.1 years. AD/MCI cases were the oldest patients (mean age of 74.5±6.6 years). The youngest participant in our study is a 40-year-old male diagnosed with ALS; the oldest are four 85-year-old participants (three males, one female); two diagnosed with AD/MCI and two with VCI. In general, sex ratios showed an over representation of males (male:female, 1.8:1.0), which was largely driven by the PD and VCI cases (3.3:1.0 and 2.0:1.0, respectively) similar to the known sex distribution of these disorders in prior population studies. In contrast, in the AD/MCI, ALS, and FTD cohorts, the male:female ratios did not differ considerably (1.5:1.0, 1.2:1.0; and 0.9:1.0, respectively). The self-reported ethnicity of the participants was predominantly Caucasian (82.3%) with some admixture. Overall, participants did not have a family history of neurodegenerative disease and were considered sporadic cases in our study as determined by participant recall, which was confirmed by the participant’s caregiver. Potential confounders such as age, sex, ethnicity and family history did not affect our study objectives or analysis.

### Quality assessment of ONDRISeq data

In total, 9 independent runs of 24 samples were processed on ONDRISeq (Table 2). All targets across the 216 DNA samples were sufficiently covered (>×30; mean coverage ×76±18; Table 2). On average, 22.8 million of 29.8 million reads passed quality filter equating to 77%. With the exception of the poorest performance run, all ONDRISeq runs had reads passed quality filter of >80%. Overall, 92.7% of all reads were mapped with 95% and 78% of reads mapped in the best and poorest performing runs, respectively. All other ONDRISeq runs had >90% of reads mapped. Of the mapped reads, 87.1% had a Phred quality score of >30 representing a base call accuracy of 99.9%. Similarly, with the exception of the poorest performing run, all ONDRISeq runs had >85% of reads with scores >Q30. Although the poorest performing run produced lower quality data compared with the other 8 ONDRISeq runs, 84.9% of its targets were covered ⩾×30 and were still analysed in our study.

Furthermore, an additional four DNA samples were extracted from brain tissue of deceased individuals. Post autopsy, sections of the brain from all four individuals were frozen for over a decade. However, we were still able to generate adequate sequence calls. Among the four samples, 96% of reads were mapped and each sample had an average coverage of ×71.

### ONDRISeq is concordant with NeuroX, TaqMan allelic discrimination assay, and Sanger sequencing

We used three independent genomic techniques, NeuroX, a genome-wide array for neurodegenerative disorders, TaqMan allelic discrimination assays, and Sanger sequencing to assess the concordance with ONDRISeq in variant detection. The NeuroX array captures known polymorphic variants within the genes represented on ONDRISeq; therefore, we evaluated whether ONDRISeq could detect the same variants as NeuroX. In doing so, we processed 115 DNA samples and ONDRISeq detected all 122 non-synonymous variants initially detected by NeuroX. Furthermore, we assessed rare and common, non-synonymous and synonymous variants called by the two platforms and observed 100% concordance between calls. Of note, there were variants detected by ONDRISeq but not included on the NeuroX array. However, there were no false negatives with ONDRISeq: all variants detected by NeuroX were also detected by ONDRISeq. Furthermore, we used a TaqMan allelic discrimination assay to genotype the same 115 DNA samples for *APOE*. Similarly, we observed 100% concordance between *APOE* genotyping calls on ONDRISeq and TaqMan.

To explore the rate of false-positive variant calls by ONDRISeq, we performed an independent concordance study for ~10% (*n*=20) of randomly selected variants from samples that were called as variants by ONDRISeq using Sanger sequencing. Similar with the results of NeuroX and TaqMan allelic discrimination assay, we observed 100% concordance in variants initially detected by ONDRISeq and validated via Sanger sequencing. Thus, there were no false positives with ONDRISeq: all variants called as variants by ONDRISeq were also called as variants by validation using Sanger sequencing.

### Clinical utility of ONDRISeq

All DNA samples were independently screened for a hexanucleotide expansion (G_4_C_2_) within *C9orf72*, a type of DNA variation that was not detectable by ONDRISeq or NeuroX. Of the 216 samples, only three (1.4%) carried an expansion within *C9orf72*, two were diagnosed with ALS and one with FTD (Table 3).

In total, we found that only 60 out of 216 samples (27.8%) were free from rare (minor allele frequency (MAF) <1%) potentially deleterious variants (missense, nonsense, frameshift, in frame insertions and/or deletions, splicing) in ONDRISeq genes (Table 4). Of the remaining 156 cases, the AD/MCI and FTD cases had the highest variant rate based on ONDRISeq (>80%), although not necessarily disease causative. In the ALS and PD cases, we identified rare coding variants in 72.7% and 71.4% of individuals, respectively. The VCI disease cohort had the lowest number of variant carriers (65%) although still significantly higher than previous reports.^16,17^ Furthermore, we tabulated the number of individuals with one, two, or three or more variants. Overall, 76 (48.7%) of 156 individuals carried one variant; 57 (36.5%) carried two variants; and 23 (14.8%) carried three or more variants (Table 4).

Among the 156 cases with potentially deleterious variants, we identified a total of 266 non-synonymous, rare variants (Table 5), including 107 (40.2%) within genes known to cause the disease with which the patient has been diagnosed (e.g., variation in an AD gene in an AD patient; Table 6). An additional 159 variants (59.8%) were found in genes that were not previously associated with the respective clinical phenotype of the patient, but within a gene responsible for another disease (e.g., variation in FTD gene in an AD patient). Of the 266 variants, which will be reported on in detail upon completion of the ONDRI study of ~600 patients, 62 (23.3%) were previously reported in HGMD and/or ClinVar; whereas 204 (76.7%) were absent from disease databases (Table 5). The majority of variants not found in disease databases were observed in FTD and PD cases (88.9% and 82.6%, respectively); whereas the majority of variants present in disease databases were observed in ALS and VCI cases (35.7% and 28%, respectively; Table 5). On average, we observed four rare variants (MAF<1%) per individual; and 1 variant per individual that met criteria set by ACMG and was considered here, as candidate variants.^15^ More rare variants were observed in individuals of African descent (16 rare variants per individual; 2 variants that met ACMG guidelines, per individual). Individuals of South Asian and Chinese origin on average carried 4.5 and 4 rare variants; and 2.5 and 2 variants meeting ACMG guidelines, respectively. These observations are likely due to ascertainment bias in the databases as they typically contain significantly more individuals of European descent than any other ethnic cohort.

Importantly, ONDRISeq is able to provide genotypes for *APOE*, which is not available through NeuroX and other arrays. In 216 cases, we did not identify a single case of *APOE E*2/*E*2 (Table 3). We identified 26 (12%) individuals who had an *APOE E*2/*E*3 genotype and 131 (60.6%) individuals who had an *APOE E*3/*E*3 genotype (Table 3). In total, 46 (21.3%) individuals were heterozygous for *APOE E*4 by possessing either an *APOE E*2/*E*4 or *APOE E*3/*E*4 genotype; whereas 13 (6.02%) individuals were homozygous for *APOE E*4 (Table 3). Not surprisingly, of the 13 *APOE E*4/*E*4 individuals, 7 (53.8%) were diagnosed with AD (Table 3).

### Case report: strong evidence of pathogenicity for APP p.Ala713Thr in AD patient

We provide an example of a single neurodegenerative disease case to demonstrate the clinical utility of ONDRISeq and our complementary bioinformatics workflow.

**Figure 1:**
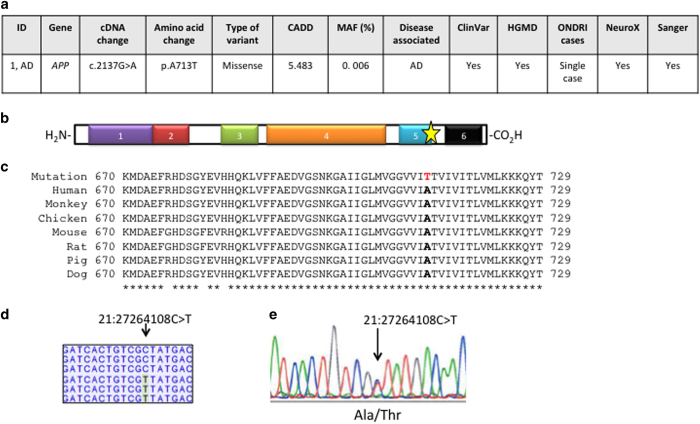
Case study: *APP* variant in AD case. (**a**) Schematic of the gene and variant discovery process in a neurodegenerative disease case. AD, Alzheimer’s disease, patient 1; *MAF was retrieved using ExAC database. (**b**) APP protein structure shown from N- to C-terminal, 1: amyloid A4 N-terminal heparin-binding domain; 2: copper-binding of amyloid precursor; 3: Kunitz/Bovine pancreatic trypsin inhibitor domain; 4: E2 domain of amyloid precursor protein; 5: beta-amyloid peptide domain; 6: beta-amyloid precursor protein C-terminus domain. The gold star represents the location of the missense variant. (**c**) Multiple alignments demonstrate high conservation of wild-type amino-acid residue p.Ala713 (in bold; the variant residue p.Thr173 is not bold) across a set of species-specific APP homologues. The asterisks below indicate fully conserved residues. (**d**) The ONDRISeq output showing heterozygosity at the position of the genetic variant, 21:27264108G>A. ONDRISeq output produced ×94 coverage. (**e**) An electropherogram showing the DNA sequence analysis of *APP* from a patient diagnosed with AD. Our reported cDNA and amino-acid positions are based on NM_000484.3 and NP_000475.1, respectively.

The patient is a 73-year-old male diagnosed with AD. We identified a heterozygous variant, namely g.11248C>T (c.2137G>A), resulting in a missense variant p.Ala713Thr in *APP*, a gene known to be associated with familial autosomal dominant AD (Figure 1a).^18^ The introduction of a polar amino acid within the beta APP domain (amino-acid residues 675–713) is predicted to affect protein function according to multiple *in silico* analyses and generated a CADD score of 5.483 (Figure 1a,b). The affected codon is also highly conserved in evolution within the APP protein when aligned to a set of diverged species within the animal kingdom (Figure 1c). The variant is very rare with MAF of 0.006% according to Exome Aggregation Consortium (ExAC) and is absent from the 1000 Genomes database and the National Heart, Lung and Blood Institute Exome Variant Server. Furthermore, the patient is the only carrier of p.Ala713Thr in APP, among the 216 samples in our study. However, the variant has been previously observed in AD cases as it is reported in both HGMD and ClinVar databases and has been previously reported in multiple publications.^19–21^ Indeed, the variant had sufficient coverage of ×94, nevertheless, we independently validated the presence of the variant using NeuroX and Sanger sequencing (Figure 1a,d,e). The patient is also homozygous for *APOE E*3/*E*3.

## Discussion

Herein, we describe a NGS based custom-designed resequencing panel to assess genes related to neurodegenerative diseases and small vessel disease. ONDRISeq is a rapid and economical diagnostic approach that screens 80 neurodegenerative genes in parallel. We have processed a total of 216 samples on ONDRISeq in 9 runs with 24 batched samples and evaluated each run using highly stringent quality assessment criteria. With ONDRISeq, we have consistently generated high-quality data and when coupled with our bioinformatics workflow, we have been able to identify rare genetic variants in >70% of patients diagnosed with one of five diseases: AD/MCI, ALS, FTD, PD, or VCI.

The ONDRISeq calls were highly reliable based on validation by three established genetic techniques: NeuroX, a rapid and economical genome-wide genotyping-based neurodegeneration array, TaqMan allelic discrimination assay, and Sanger sequencing. Although NeuroX is able to genotype >250,000 SNPs, the advantage of ONDRISeq is that it is sequencing-based and is able to detect novel variants.^22^ This way, we can agnostically screen individuals for any novel or known variants within the 80 neurodegenerative genes. Furthermore, although the TaqMan allelic discrimination assay is a rapid genotyping approach, specific probes have to be designed for all SNPs of interest, becoming ultimately costly and inefficient. Also, unlike Sanger sequencing, ONDRISeq is rapid, efficient, and economical. Following library preparation, we are able to analyse the genetic data for 24 samples in <30 h.

We calculated the cost of sequencing 80 genes using standard Sanger sequencing. The total size of ONDRISeq is 971,388 base pairs, which can be processed via ~1,943 PCR reactions (estimation of 500 base pairs per reaction). Had we processed the sequencing reactions in bulk, the cost per sample for Sanger sequencing would have been $38,860 CND per individual. Using NGS-based approaches like WGS or WES with adequate coverage, the price still remains relatively high at $1,400 and $700 CND, respectively (prices based on The Centre for Applied Genomics, Toronto, ON, Canada; www.tcag.ca). Conversely, through strategic cost management we were able to bring our overall expenditures to a highly competitive price of $340 per sample—a reduction of >99% in cost of Sanger sequencing; a >75% reduction relative to WGS, and >50% reduction relative to WES.

Despite its efficiency and rapidity, there are still some limitations with ONDRISeq. First, it can only capture variants within the selected 80 genes, which prevents the discovery of novel disease loci. However, its custom design allows its genetic content to be altered to include novel genomic regions of interest. Second, ONDRISeq is unable to capture multi-nucleotide repeat expansions in genes, a limitation across all NGS platforms.^23^ Many neurological diseases such as Huntington’s disease, myotonic dystrophy, Friedreich’s ataxia, Fragile X syndrome, and a subset of spinocerebellar ataxias arising due to multi-nucleotide repeat expansions cannot be detected with current NGS methodologies.^24,25^ More recently, a hexanucleotide (G_4_C_2_) repeat expansion in *C9orf72* has been observed in familial and sporadic ALS and FTD cases, and very rarely in PD cases.^26–28^ Since its discovery in 2011, it has been one of the top investigated genes as both a diagnostic marker and a therapeutic target.^26,27^ *C9orf72* alleles can range from 2 to 20 repeats, which are common in the healthy population and are likely benign; 20 to few hundred repeats, which confer risk; or more than few hundred repeats and are pathogenic.^7,29^ As such, we independently examined all individuals in our cohort for the *C9orf72* expansion using: (1) an amplicon length PCR analysis and (2) a repeat primed PCR analysis. In doing so, we identified that 1.4% of the participants were carriers of a *C9orf72* repeat expansion.

Despite these limitations and the complex heterogeneity in the five neurodegenerative diseases that are being assessed with ONDRISeq, we were able to capture rare variants with a probable, but not certain disease association based on allele frequency in the general population and the predictive score of multiple *in silico* software in 72.2% of cases. As the aetiology of neurodegenerative diseases is often heterogeneous and multiple factors (e.g., genetics, dietary intake, traumatic brain injury, serious infections or toxin exposure) can confer risk to disease onset, we intend to functionally validate the genetic variants, especially the novel variants, to determine their effect size and contribution to disease. Of particular interest are variants in genes with multiple disease associations as they may provide clues on the potential for development of therapy to treat symptoms common across all five neurodegenerative diseases

## Materials and methods

### Design of ONDRISeq

Using multiple databases, we catalogued literature of neurodegeneration genetic studies. We surveyed 25 content experts (professors, scientists and clinicians within ONDRI) in molecular genetics of neurodegeneration, and used their consensus opinions to select 80 genes within the human genome that were involved in one or more of the five neurodegenerative disorders under study (Table 6). Most genes were selected based on being implicated in neurodegeneration from human genetic studies; however, some of the genes were added based on pathway analysis. Furthermore, some genes were omitted from the ONDRISeq panel due to technical challenges, such as those involving repetitive sequence regions in the genome. This was the case for *GBA* gene, which is associated with an increased risk of developing PD^30^ and will thereby be assessed in separate sequencing experiments. Another gene that was omitted from the panel is *C9orf72*, which contains a repeat expansion and was therefore assessed with a separate genotyping assay as described in subsequent sections.

We designed a composition for detecting variants in the protein-coding regions of 80 genes summing to 1,649 targets. The 80 genes selected have a total target size of 972,388 base pairs. Using the NGS chemistry Nextera Rapid Custom Capture (Illumina, San Diego, CA, USA), we designed a total of 14,510 target specific probes that are each ~80 base pairs in length. For regions that were difficult to sequence, we incorporated additional probes to ensure sufficient coverage during sequencing thereby producing fewer false discoveries. Chromosome scaffold coordinates were obtained from the University of California Santa Cruz Genome Browser using the February 2009 GRCh37/hg19 genome build and were submitted to the Illumina Online Design Studio (Illumina).

### Sample collection and DNA isolation

Blood samples were collected from 216 subjects following appropriate and informed consent in accordance with the Research Ethics Board at Parkwood Hospital (London, Ontario, Canada); London Health Sciences Centre (London, Ontario, Canada); Sunnybrook Health Sciences Centre (Toronto, Ontario, Canada); University Health Network-Toronto Western Hospital (Toronto, Ontario, Canada); St Michael's Hospital (Toronto, Ontario, Canada); Centre for Addiction and Mental Health (Toronto, Ontario, Canada); Baycrest Centre for Geriatric Care (Toronto, Ontario, Canada); Hamilton General Hospital (Hamilton, Ontario, Canada); McMaster (Hamilton, Ontario, Canada); Elizabeth Bruyère Hospital (Ottawa, Ontario, Canada); and The Ottawa Hospital (Ottawa, Ontario, Canada).

All clinical diagnoses were supplied by each patient’s healthcare provider in accordance with the criteria from the general ONDRI protocol^31^. DNA was isolated from 4 to 8 ml of blood collected from every participant using the Gentra Puregene Blood kit (Qiagen, Venlo, The Netherlands) according to the manufacturer’s instructions. DNA quality and concentration were initially measured by NanoDrop-1000 Spectrophotometer (Thermo Fisher Scientific, Waltham, MA, USA) and followed by subsequent serial dilutions to obtain ~5 ng/μl. Qubit 2.0 fluorometer technology (Invitrogen, Carlsbad, CA, USA) was then used to measure lower concentrations of DNA at a higher sensitivity.

### Library preparation

Libraries were prepared in house using the Nextera Rapid Custom Capture Enrichment kit in accordance with manufacturer’s instructions. DNA samples were processed in sets of 12. DNA samples were fragmented followed by ligation of Nextera Custom Enrichment Kit-specific adapters, amplified via PCR using unique sample barcodes, equimolar pooled, and hybridised to target probes (two cycles of 18 h each). Samples were then amplified again to ensure specificity and greater DNA yield. A small aliquot of each library was analysed using the Agilent 2100 BioAnalyzer (Agilent Technologies, Palo Alto, CA, USA) to ensure adequate yield. The quantity and quality of the final libraries were measured using the KAPA quantitative PCR library quantification kit (KAPA Biosystems, Woburn, MA, USA) using the ViiA 7 Real-Time PCR System (Thermo Fisher Scientific).

### Next generation sequencing

All samples were sequenced on the Illumina MiSeq Personal Genome Sequencer (Illumina) using the MiSeq Reagent Kit v3 in accordance with manufacturer’s instructions. Indexed samples were pooled in equimolar ratios of 500 ng. Once combined, 16 pM of denatured pooled library was loaded on to a standard flow-cell on the Illumina MiSeq Personal Sequencer using 2×150 bp paired-end chemistry. Viral PhiX DNA was added as a positive control to ensure sequencer performance. Sequencing quality control was assessed using multiple parameters in Illumina MiSeq Reporter and visualised either in Illumina BaseSpace or locally using Illumina Sequencing Analysis Viewer.

### Variant calling

After demultiplexing and adapter trimming, FASTQ files were aligned to the consensus human genome sequence build GRCh37/hg19 using a customised workflow within CLC Bio Genomics Workbench v6.5 (CLC Bio, Aarhus, Denmark) as previously described.^32^ Similarly, variant annotation was performed using ANNOVAR as previously described^32^ with additional databases such as CADD^33^, HGMD (release 2015.1.), ClinVar^34^, ExAC^35^ and our own in-house databases.

### *APOE* genotyping

Furthermore, using ONDRISeq, in addition to screening all samples for variants within *APOE*, we genotyped all individuals for the *APOE* risk alleles rs429358(CT) and rs7412(CT). The combination of both individual alleles determines the *APOE* genotype and is known to be one of the major genetic risk factors for late onset AD.^18^ If there are no deletions at these loci, six potential *APOE* allele combinations are possible (2 alleles×3 possible genotypes): (1) *E*2/*E*2; (2) *E*3/*E*2; (3) *E*4/*E*2; (4) *E*3/*E*3; (5) *E*4/*E*3; and (6) *E*4/*E*4, the latter of which is associated with up to an 11x increased risk in developing AD.^18,36^

### Variant classification and prioritisation

In general, we followed the guidelines for the interpretation of sequence variants proposed by the American College of Medical Genetics and Genomics and the Association for Molecular Pathology^37^. We screened for rare variants, which in our study were considered to be variants with MAF<1% based on 1000 Genomes, NHLBI Exome Sequencing Project, and the ExAC databases. Among rare variants, we investigated whether there were any non-synonymous changes (nucleotide substitutions, insertions or deletions) that resulted in missense, nonsense, splicing or frameshift variation. Variants were also assessed *in silico* using a compilation of prediction programs: PolyPhen-2, SIFT and CADD. HGMD and ClinVar were also integrated to determine the novelty or recurrence of any genetic variation with a specific disease state. More specifically, we were interested in determining how many variants were previously deposited into disease databases. In our study, variants were marked as clinically relevant if they were rare, resulted in non-synonymous changes, were previously observed in individuals with the same disease state, and had values consistent with ‘disease-causing’ based on prediction outcomes of PolyPhen-2, SIFT and CADD, as recommended by ACMG Standards and Guidelines. Importantly, we grouped the variants according to the categories set forth by the ACMG Standards and Guidelines. Alternatively, variants classified here as variants with uncertain clinical significance, were variants that were not reported in disease databases and were observed in genes that are not typically causative of the disease in which the individual is diagnosed with, as represented on Table 6. For example, a variant in a gene that is associated with ALS in a patient diagnosed with VCI. Finally, all ONDRI samples were compared with each other to resolve whether any variants were observed in multiple individuals with the same disease diagnosis. We used this approach to also determine whether the same variant(s) was present in a large subset of ONDRI samples and therefore, was more likely to be an artifact of sequencing or alignment.

### Variant validation

To validate variants detected by ONDRISeq, we used three independent genotyping techniques, namely (1) NeuroX, which is an array of specific genotypes that confer risk to several neurodegenerative disease phenotypes;^22^ (2) TaqMan allelic discrimination; and (3) Sanger sequencing. We processed 115 samples on the NeuroX and the TaqMan allelic discrimination assay and determined the concordance rate between each assay and ONDRISeq. We also randomly selected ~10% of variants to genotype using Sanger sequencing to determine the occurrence if any, of false positives. To test for true negatives, we used DNA from four individuals who were diagnosed with ALS. These individuals were previously tested for genetic variation within *SOD1* with no variants identified. Similarly, we did not identify any variants in *SOD1* using ONDRISeq. The NeuroX genotyping, TaqMan allelic discrimination assay, and the prior *SOD1* testing of ALS samples was performed independently without knowledge of the variants generated using ONDRISeq. Furthermore, different lab personnel completed each validation step to ensure objectivity when evaluating concordance of results.

### Variant validation 1: NeuroX

DNA samples were genotyped on NeuroX exome array (Illumina) according to manufacturer’s instructions. NeuroX data were loaded to GenomeStudio (Illumina) and all markers were clustered using the default Gen Call threshold (0.15); duplicate samples (*N*=2) revealed identical genotypes for all markers with available genotypes (*N*=268,399).^22^ Genotypes were converted to PLINK input files, and allele frequencies were calculated. In total, the 115 samples revealed 71,714 polymorphic autosomal markers including 43,129 exonic and 216 splicing variants; among them 39,390 polymorphisms were non-synonymous, as well as 423 stop-gain and 32 stop-loss variants, according to ANNOVAR analyses.^22^ Average sample call rate was 99.6%, indicating high genotype quality.

Next, 1,047 polymorphic markers, which included 252 exonic variants (229 nonsynonymous and 1 splicing) within the 80 genes of the ONDRISeq targeted sequencing panel, were further processed by removing all noncoding, synonymous and common variants with MAF>1% in any database of 1000Genomes (1000g2014oct_all), Exome Variant Server (esp6500si_all) and ExAC. Variants overlapping segmental duplications were also excluded to avoid possible genotyping error. The remaining variants were filtered to those predicted to have a potential damaging effect on protein function, according to either PolyPhen-2 or SIFT analyses implemented in ANNOVAR.

### Variant validation 2: TaqMan allelic discrimination

*APOE* SNP genotyping was performed using the TaqMan allelic discrimination assay for 115 samples on the 7900HT Fast Real-Time PCR System (Life Technologies, Foster City, CA, USA), and genotypes were identified using automated software (SDS 2.3; Life Technologies). Two TaqMan assays were used to determine the *APOE* genotype, namely (1) C_3084793_20 (rs429358: *APOE* codon 112) and (2) C_904973_10 (rs7412; *APOE* codon 158).

### Variant validation 3: Sanger sequencing

Briefly, genomic DNA from the samples was first amplified via PCR, cleaned and purified, and sequenced at the London Regional Genomics Centre. Electropherograms produced were analysed using Applied Biosystems (ABI) SeqScape Software version 2.6 (Thermo Fischer Scientific, Waltham, MA, USA) with the reference sequence of each gene obtained from NCBI GenBank database.

### Variant validation 4: *SOD1* testing

Screening for genetic variants in the *SOD1* gene was performed by PCR followed by standard Sanger sequencing methods, on DNA from four individuals diagnosed with ALS. These steps were performed in other research laboratories prior to this study. Using ONDRISeq, we sequenced DNA from these four individuals to determine whether there were any *SOD1* genetic variants. This step allows us to evaluate any true-/false-negative discoveries.

### *C9orf72* genotyping

All participants were genotyped for the G_4_C_2_-expansion in *C9orf72* using a two-step method: (1) amplicon length analysis and (2) repeat-primed PCR. Experimental procedures are described elsewhere.^28^

### Statistical analysis

The Student’s *t*-test was used to determine the significance of the difference among patient characteristics within the different neurodegenerative disease cohorts, where appropriate.

## Figures and Tables

**Table 1:** Patient demographics

*Disease ID*	*Cases*	*Mean age (years±s.d.)*	*Min age (years)*	*Max age (years)*	*Male:female*	*Self-reported ethnicity as Caucasian (%)*	*Family history of neurodegeneration?*
Total	216	69.4±7.8	40	85	140:76	82.3	Mainly sporadic
Alzheimer’s disease/mild cognitive impairment	40 (18.5%)	74.5±6.6	59	85	24:16	93.3	
Amyotrophic lateral sclerosis	22 (10.2%)	61.9±9.1	40	77	12:10	67.9	
Frontotemporal dementia	21 (9.8%)	68.8±6.6	55	79	10:11	82.6	
Parkinson’s disease	56 (25.9%)	68.0±5.9	57	82	43:13	83.8	
Vascular cognitive impairment	77 (35.6%)	70.2±7.4	55	85	51:26	84.0	

**Table 2:** Quality control metrics for sequencing runs on ONDRISeq

*Parameters*	*Mean (±s.d.)*	*Best performance*	*Poorest performance*
Cluster density (×10^3^/mm^2^)	1433.6 (±165)	1320	1835
Target size (bp)	971,388	971,388	971,388
Total reads (×10^6^)	29.8 (±2.5)	29.1	35.6
Reads PF (×10^6^)	22.8 (±0.9)	24.1	22.1
Reads PF (%)	77 (±5.8)	83	62
Targets bases ⩾30 (%)	92.0%	95.3	84.9
Mean target coverage	76 (±18)		
Max target coverage	259		
Min target coverage	0		

Abbreviation: PF, passed quality filter.

Mean of 9 runs. Blank spaces represent ‘not applicable’.

**Table 3:** Other risk variants identified in a cohort of 216 disease cases

*Disease ID*	*C9orf72 expansion carriers*	*APOE E2/E2 genotype*	*APOE E2/E3 genotype*	*APOE E2/E4 genotype*	*APOE E3/E3 genotype*	*APOE E3/E4 genotype*	*APOE E4/E4 genotype*
Total (*n*=216)	3 (1.40%)	0 (0.00%)	26 (12.0%)	1 (0.46%)	131 (60.6%)	45 (20.8%)	13 (6.02%)
AD/MCI (*n*=40)	0 (0.00%)	0 (0.00%)	1 (2.50%)	0 (0.00%)	17 (42.5%)	15 (37.5%)	7 (17.5%)
ALS (*n*=22)	2 (9.09%)	0 (0.00%)	4 (18.2%)	0 (0.00%)	12 (54.5%)	6 (27.3%)	0 (0.00%)
FTD (*n*=21)	1 (4.76%)	0 (0.00%)	1 (4.76%)	0 (0.00%)	13 (61.9%)	5 (23.8%)	2 (9.52%)
PD (*n*=56)	0 (0.00%)	0 (0.00%)	10 (17.9%)	1 (1.79%)	39 (69.6%)	5 (8.90%)	1 (1.79%)
VCI (*n*=77)	0 (0.00%)	0 (0.00%)	10 (13.0%)	0 (0.00%)	50 (64.9%)	14 (18.2%)	3 (3.90%)

Abbreviations: AD/MCI, Alzheimer’s disease/mild cognitive impairment; ALS, amyotrophic lateral sclerosis; FTD, frontotemporal dementia; PD, Parkinson’s disease; VCI, vascular cognitive impairment.

**Table 4:** Diagnostic yield of ONDRISeq in a cohort of 216 disease cases

*Disease ID*	*Individuals without any variants*	*Individuals with variants*	*Individuals with 1 variants*	*Individuals with 2 variants*	*Individuals with ⩾3 variants*
Total (*n*=216)	60 (27.8%)	156 (72.2%)	76 (48.7%)	57 (36.5%)	23 (14.8%)
AD/MCI (*n*=40)	7 (17.5%)	33 (82.5%)	18 (54.5%)	10 (30.3%)	5 (15.2%)
ALS (*n*=22)	6 (27.3%)	16 (72.7%)	6 (37.5%)	8 (50.0%)	2 (12.5%)
FTD (*n*=21)	4 (19.0%)	17 (81.0%)	9 (52.9%)	7 (41.2%)	1 (5.9%)
PD (*n*=56)	16 (28.6%)	40 (71.4%)	22 (55.0%)	13 (32.5%)	5 (12.5%)
VCI (*n*=77)	27 (35.1%)	50 (64.9%)	21 (42.0%)	19 (38.0%)	10 (20%)

Abbreviations: AD/MCI, Alzheimer’s disease/mild cognitive impairment; ALS, amyotrophic lateral sclerosis; FTD, frontotemporal dementia; PD, parkinson’s disease; VCI, vascular cognitive impairment.

Variant criteria were based on non-synonymous, rare variants (<1% in ExAC). The variants here and in Table 5 are the same but tabulated differently.

**Table 5:** Variants identified in a cohort of 216 disease cases as detected by ONDRISeq

*Disease ID*	*Individuals with variants*	*ONDRISeq variants*	*Variants in disease gene as diagnosed*	*Variants in other ONDRISeq disease genes*	*Variants in disease databases*	*Variants not found in disease databases*
Total (*n*=216)	156 (72.2%)	266	107 (40.2%)	159 (59.8%)	62 (23.3%)	204 (76.7%)
AD/MCI (*n*=40)	33 (82.5%)	55	19 (34.5%)	36 (65.5%)	12 (21.8%)	43 (78.2%)
ALS (*n*=22)	16 (72.7%)	28	17 (60.7%)	11 (39.2%)	10 (35.7%)	18 (64.3%)
FTD (*n*=21)	17 (81.0%)	27	12 (44.4%)	15 (55.6%)	3 (11.1%)	24 (88.9%)
PD (*n*=56)	40 (71.4%)	63	31 (49.2%)	32 (50.8%)	11 (17.5%)	52 (82.6%)
VCI (*n*=77)	50 (64.9%)	93	28 (30.1%)	65 (69.9%)	26 (28.0%)	67 (72.0%)

Abbreviations: AD/MCI, Alzheimer’s disease/mild cognitive impairment; ALS, amyotrophic lateral sclerosis; FTD, frontotemporal dementia; PD, Parkinson’s disease; VCI, vascular cognitive impairment.

‘ONDRISeq variants refers to the total number of variants identified in each disease cohort or the total number of neurodegenerative disease cases. ‘Variants in disease gene as diagnosed’ refers to variants in genes known to cause the disease the patient is diagnosed with. ‘Variants in other ONDRI disease genes’ refers to variants identified in genes that are not typically associated with the disease the patient is diagnosed with as categorised on the ONDRISeq gene panel. ‘Variants in disease databases’ were classified as variants present within HGMD or ClinVar. Similarly, ‘Variants not found in disease databases’ were classified as variants absent from HGMD or ClinVar. Values in parentheses in columns 4-7 were calculated by dividing the values by the total ONDRISeq variants listed in column 3. The variants in Table 4 and here are the same but tabulated differently.

**Table 6:** Genes associated with amyotrophic lateral sclerosis, frontotemporal dementia, Alzheimer’s disease, Parkinson’s disease, or vascular cognitive impairment as represented on the ONDRISeq targeted resequencing panel

*Gene*	*Chromosomal location*	*Affected protein*	*Associated phenotype*	*Mode of inheritance*	*OMIM numbers (locus, phenotype)*
*Amyotrophic lateral sclerosis/frontotemporal dementia*					
*ALS2*	2q33.1	Alsin	ALS2	AR (HZ), juvenile onset	606352, 205100
*ANG*	14q11.2	Angiogenin	ALS9	ADm, late onset	105850, 611895
*ARHGEF28*	5q13.2	Rho guanine nucleotide exchange factor 28	ALS and FTD	AR (HZ) and ADm, late onset	612790, PMID: 23286752 (phenotype not updated on OMIM)
*ATXN2*	12q24.12	Ataxin 2	ALS13	ADm, late onset	601517, 183090
*CENPV*	17p11.2	Centromere protein V	ALS	Genetic association, late onset	608139, PMID: 22959728 (phenotype not updated on OMIM)
*CHMP2B*	3p11.2	CHMP family member 2B	ALS17, FTD	ADm, late onset	609512, 614696
*DAO*	12q24.11	D-amino acid oxidase	ALS, schizophrenia	ADm, late onset	124050, 105400, 181500
*DCTN1*	2p13.1	Dynactin 1	ALS, HMN7B, Perry syndrome	ADm, late onset	601143, 105400, 607641, 168605
*FIG4*	6q21	FIG4 homologue, SAC1 lipid phosphatase domain containing	ALS11, CMT disease, YV syndrome	ADm, late onset; AR (HZ and CH), infantile onset; AR (HZ and CH), infantile onset	609390, 612577, 611228, 216340
*FUS*	16p11.2	Fused in sarcoma	ALS6, FTD, HET4	AR (HZ), ADm, late onset	137070, 608030, 614782
*GRN*	17q21.31	Granulin precursor	FTD, NCL	ADm, late onset; AR (HZ), juvenile onset	138945, 607485, 614706
*HNRNPA1*	12q13.13	Heterogeneous nuclear ribonucleoprotein A1	ALS20, inclusion body myopathy with early-onset Paget disease with/without FTD 3	ADm, late onset; ADm, early onset	164017, 615426, 615424
*HNRNPA2B1*	7p15.2	Heterogeneous nuclear ribonucleoprotein A2/B1	Inclusion body myopathy with early-onset Paget disease with/without FTD 2	ADm, early onset	600124, 615422
*MAPT/STH*	17q21.31	Microtubule-associated protein tau	ALS, FTD with parkinsonism, PD, AD, Pick disease, supranuclear palsy, tauopathy	ADm, late and early onset	157140, 105400, 600274, 168600, 104300, 172700, 601104, 260540
*NEFH*	22q12.2	Neurofilament protein, heavy polypeptide	ALS1	ADm, late onset	162230, 105400
*OPTN*	10p13	Optineurin	ALS12, glaucoma	AR (HZ) and AD, early onset	602432, 613435, 606657
*PFN1*	17p13.2	Profilin 1	ALS18	ADm, earlier onset	176610, 614808
*PNPLA6*	19p13.2	Patatin-like phospholipase domain-containing protein 6	Spastic paraplegia, Boucher-Neuhauser syndrome	AR (HZ and CH), early onset	603197, 612020, 215470
*PRPH*	12q13.12	Peripherin	ALS1	ADm, late onset	170710, 105400
*SETX*	9q34.13	Senataxin	ALS4, spinocerebellar ataxia 1	ADm and AR, juvenile onset	608465, 602433, 606002
*SIGMAR1*	9p13.3	Sigma nonopioid intracellular receptor 1	ALS16, FTD	AR (HZ); ADm, early onset	601978, 614373, 105550
*SOD1*	21q22.11	Superoxide dismutase 1	ALS1	AR (HZ and CH), ADm, age of onset varies from 6–94 years old	147450, 105400
*SQSTM1*	5q35.3	Sequestosome 1	Paget disease of bone	ADm, late onset	601530, 167250
*TAF15*	17q12	TAF15 RNA polymerase II, TATA box-binding protein-associated factor	Chondrosarcoma		601574, 612237
*TARDBP*	1p36.22	Tar DNA-binding protein	ALS10, FTD	ADm, late onset	605078, 612069
*UBQLN2*	Xp11.21	Ubiquilin 2	ALS15, FTD	X-linked, juvenile and late onset	300264, 300857
*UNC13A*	19p13.11	Unc-13 homolog A (*C. elegans*)	ALS	Genetic association, late onset	609894, PMID: 22921269 (phenotype not updated on OMIM)
*VAPB*	20q13.33	Vesicle-associated membrane protein (VAMP)-associated protein B and C	ALS, spinal muscular atrophy (Finkel type)	ADm, early and late onset	605704, 608627, 182980
*VCP*	9p13.3	Valosin-containing protein	ALS14, FTD, inclusion body myopathy with early-onset Paget disease with/without FTD 1	ADm, early onset	601023, 613954, 167320
					
*Alzheimer’s disease/mild cognitive impairment*					
*ABCA7*	19p13.3	ATP-binding cassette, subfamily a, member 7	AD	Genetic association, late onset	605414, 104300
*APOE*	19q13.32	Apolipoprotein E	AD2, lipoprotein glomerulopathy, sea-blue hystiocyte disease, macular degeneration	ACD, ADm, AR (HZ and CH), late onset	107741, 104310, 611771, 269600, 603075
*APP*	21q21.3	Amyloid beta A4 precursor protein	AD 1, cerebral amyloid angiopathy	ADm and AR (HZ), early and late onset	104760, 104300, 605714
*BIN1*	2q14.3	Bridging integrator 1	AD	Genetic association, late onset	601248, PMID: 25365775 (phenotype not updated on OMIM)
*CD2AP*	6p12.3	CD2-associated protein	AD	Genetic association, late onset	604241, PMID: 25092125 (phenotype not updated on OMIM)
*CD33*	19q13.41	CD33 antigen	AD	Genetic association, late onset	159590, PMID: 23982747 (phenotype not updated on OMIM)
*CLU*	8p21.1	Clusterin	AD	Genetic association, late onset	185430, PMID: 25189118 (phenotype not updated on OMIM)
*CR1*	1q32.2	Complement component receptor 1	AD	Genetic association, late onset	120620, PMID: 25022885 (phenotype not updated on OMIM)
*CSF1R*	5q32	Colony-stimulating factor 1 receptor	HDLS with dementia	ADm, early and late onset	164770, 221820
*DNMT1*	19p13.2	DNA methyltransferase 1	HSN1E with dementia	ADm, early onset dementia	126375, 614116
*ITM2B*	13q14.2	Integral membrane protein 2B	Dementia	ADm, early and late onset	603904, 176500, 117300
*MS4A4E*	11q12.2	Membrane-spanning 4-domains, subfamily A, member 4E	AD	Genetic association, late onset	608401, PMID: 21460840 (phenotype not updated on OMIM)
*MS4A6A*	11q12.2	Membrane-spanning 4-domains, subfamily A, member 6A	AD	Genetic association, late onset	606548, PMID: 21460840 (phenotype not updated on OMIM)
*PICALM*	11q14.2	Phosphatidylinositol-binding clathrin assembly protein	AD	Genetic association, late onset	603025, PMID: 24613704 (phenotype not updated on OMIM)
*PLD3*	19q13.2	Phospholipase D family, member 3	AD19	Genetic association, late onset	615698, 615711
*PSEN1*	14q24.2	Presenilin 1	AD3, dilated cardiomyopathy, FTD, Pick disease, acne inversa	ADm, early onset	104311, 607822, 613694, 600274, 172700, 613737
*PRNP*	20p13	Prion protein	Dementia	ADm, early onset	176640, 606688
*PSEN2*	1q32.13	Presenilin 2	AD4, dilated cardiomyopathy	ADm, early onset	600759, 606889, 613697
*SORL1*	11q24.1	Sortilin-related receptor	AD	ADm, combined gene burden, late onset	602005, 104300; PMID: 25382023 (phenotype not updated on OMIM)
*TREM2*	6p21.1	Triggering receptor expressed on myeloid cells 2	AD Nasu-Hakola disease (dementia and psychotic symptoms)	Genetic association, late onset	605086, PMID: 25596843 (phenotype not updated on OMIM), 221770
*TYROBP*	19q13.12	Tyro protein tyrosine kinase-binding protein	Nasu–Hakola disease (dementia and psychotic symptoms)	AR (HZ), juvenile onset	604142, 221770
					
*Parkinson’s disease*					
*ADH1C*	4q23	Alcohol dehydrogenase 1C, gamma polypeptide	PD, alcohol dependence protection	Genetic association, late onset	103730, 168600, 103780
*ATP13A2 (PARK9)*	1p36.13	ATPase, type 13A2	PD, ceroid lipofuscinosis, dementia	Genetic association, early onset and late onset	610513, 606693
*DNAJC13*	3q22.1	DNAJ/HSP40 homolog, subfamily C, member 13	PD	ADm, late onset	614334, PMID: 25330418 (phenotype not updated on OMIM)
*EIF4G1*	3q27.1	Eukaryotic translation initiation factor 4-gamma	PD18	ADm, late onset	600495, 614251
*FBXO7*	22q12.3	F-box only protein 7	PD15	AR (HZ and CH), early onset	605648, 260300
*GAK*	4p16.3	Cyclin G-associated kinase	PD	Genetic association, late onset	602052, PMID: 21258085 (phenotype not updated on OMIM)
*GCH1*	14q22.2	GTP cyclohydrolase I	PD, dystonia	Genetic association, early onset	600225, 128230
*GIGYF2*	2q37.1	GRB10-interacting GYP protein 2	PD11	Genetic association, early and late onset	612003, 607688
*HTRA2*	2p13.1	HTRA serine peptidase 2	PD13	ADm and genetic association, early and late onset	606441, 610297
*LRRK2*	12q12	Leucine-rich repeat kinase 2	PD8	ADm and genetic association, early and late onset	609007, 607060
*MC1R*	16q24.3	Melanocortin 1 receptor	PD; melanoma, UV induced skin damage	Genetic association, late onset	155555, 613099, 266300, 168600
*NR4A2*	2q24.1	Nuclear receptor subfamily 4, group A, member 2	PD	Genetic association, late onset	601828, 168600
*PANK2*	20p13	Pantothenate kinase 2	Neurodegeneration	AR (HZ and CH), early onset	606157, 234200
*PARK2 (PRKN)*	6q26	Parkin	PD2	AR (HZ and CH), juvenile onset; heterozygotes have late onset	602544, 600116
*PARK7(DJ1)*	1p36.23	Oncogene DJ1	PD7	AR (HZ and CH), early onset	602533, 606324
*PARL*	3q27.1	Presenilin-associated rhomboid-like protein	PD (based on biological mechanisms, no linkage confirmed)	NA	607858, PMID: 21355049 (phenotype not updated on OMIM)
*PINK1*	1p36.12	Pten-induced putative kinase 1	PD6	AR (HZ and CH), ADm, early onset	608309, 605909
*PLA2G6*	22q13.1	Phospholipase A2, group VI	PD14, NBIA2A, NBIA2B	AR (HZ and CH), early and late onset	603604, 612953, 256600, 610217
*PM20D1*	1q32	Peptidase M20 domain containing 1	PD16	Genetic association, late onset	Locus ID not available on OMIM, 613164
*RAB7L1*	1q32.1	RAB7-like 1	PD	Genetic association, late onset	603949, PMID: 25040112 (phenotype not updated on OMIM)
*SNCA*	4q22.1	Alpha-synuclein	PD1, PD4, LBD	ADm, early onset	163890, 168601, 605543, 127750
*UCHL1*	4p13	Ubiquitin carboxyl-terminal esterase L1	PD5, neurodegeneration with optic atrophy	ADm, AR (HZ), juvenile-onset	191342, 613643, 615491
*VPS35*	16q11.2	Vacuolar protein sorting 35	PD17	ADm, early and late onset	601501, 614203
					
*Vascular cognitive impairment*					
*ABCC6*	16p13.11	ATP-binding cassette, subfamily C, member 6	Arterial calcification; pseudoxanthoma elasticum; pseudoxanthoma elasticum forme fruste	AR (HZ), infantile onset; AR; ADm	603234, 614473, 264800, 177850
*COL4A1*	13q34	Collagen type IV, alpha-1	Angiopathy, brain small vessel disease, porencephaly 1, intracerebral haemorrhage susceptibility	ADm, infantile onset	120130, 611773, 607595, 175780, 614519
*COL4A2*	13q34	Collagen type IV, alpha-2	Porencephaly 2, intracerebral haemorrhage susceptibility	ADm, infantile onset	120090, 614483, 614519
*HTRA1*	10q26.13	HTRA serine peptidase 1	CARASIL syndrome, macular degeneration	AR (HZ), early onset	602194, 600142, 610149
*NOTCH3*	19p13.12	Notch homology protein 3	Infantile myofibromatosis 2, CADASIL	ADm, early onset	600276, 615293, 125310
*SAMHD1*	20q11.23	SAM domain and HD domain 1	Aicardi-Goutieres syndrome 5, Chilblain lupus 2	AR (HZ and CH), AD, infantile onset	606754, 612954, 614415
*TREX1*	3p21.31	3-prime repair exonuclease 1	Aicardi-Goutieres syndrome 1, Chilblain lupus, Vasculopathy, retinal, with cerebral leukodystrophy	AR (HZ and CH), juvenile onset, AD, AD	606609, 225750, 610448, 192315

Abbreviations: ACD, autosomal co-dominant; AD, Alzheimer’s disease; ADm, autosomal dominant; ALS, amyotrophic lateral sclerosis; AR, autosomal recessive; CARASIL syndrome, cerebral autosomal recessive arteriopathy with subcortical infarcts and leukoencephalopathy; CADASIL, cerebral autosomal dominant arteriopathy with subcortical infarcts and leukoencephalopathy; CH, compound heterozygous; CMT disease, Charcot-Marie-Tooth disease; FTD, frontotemporal dementia; HDLS, leukoencephalopathy, diffuse hereditary, with spheroids; HET4, hereditary essential tremor, 4; HMN7B, neuropathy, distal hereditary motor, type VIIB; HSN1E, hereditary sensory neuropathy type 1E; HZ, homozygous; LBD, Lewy body dementia; NCL, neuronal ceroid-lipofuscinoses; NBIA2A, neurodegeneration with brain iron accumulation 2A; NBIA2B, neurodegeneration with brain iron accumulation 2B; PD, Parkinson’s disease; OMIM, Online Mendelian Inheritance in Man; PMID, PubMed identification; YV syndrome, Yunis–Varon syndrome.

Age of onset was classified as ‘late onset’ if greater than 65 years of age.
